# Alterations of ocular surface parameters in patients with obstructive sleep apnea syndrome

**DOI:** 10.3389/fmed.2023.1220104

**Published:** 2023-09-20

**Authors:** Linlin Hao, Qingfen Tian, Shaohua Liu, Zhe Xu, Lixia Yang

**Affiliations:** ^1^Department of Ophthalmology, The Second Hospital of Shandong University, Cheeloo College of Medicine, Shandong University, Jinan, China; ^2^Department of Otolaryngology, Jinan Second People’s Hospital of Shandong University, Cheeloo College of Medicine, Shandong University, Jinan, China

**Keywords:** obstructive sleep apnea syndrome (OSAS), ocular surface, dry eye, BMI, PBR

## Abstract

**Purpose:**

This study aimed to evaluate changes in ocular surface parameters among obstructive sleep apnea syndrome (OSAS) patients.

**Methods:**

44 healthy volunteers (88 eyes) and 27 OSAS patients (54 eyes) were recruited in our cross-sectional study. 14 patients were classified as mild&moderate OSAS, and 13 patients were classified as severe OSAS. For evaluating the ocular surface, the following tests were conducted: the height of tear meniscus (TMH), first non-invasive tear break-up time (FNITBUT), mean non-invasive tear break-up time (MNITBUT), the score of Meibomian gland dropout area (Meiboscore), the tear test of anesthesia-free Schirmer I (SIT), corneal fluorescein staining (CFS), partial blinks rate (PBR), the lipid layer thickness (LLT), ocular surface disease index (OSDI). The results obtained from the study were analyzed and compared among the groups.

**Results:**

FNITBUT, MNITBUT, and TMH were lower. OSDI, CFS, Meiboscore and PBR were higher in the OSAS group than those in the control group. The mild&moderate as well as the severe OSAS subgroups had statistically significantly lower TMH, and higher OSDI and PBR than the control group. Meanwhile, we found there were no significant differences between two OSAS subgroups. CFS was higher in the severe OSAS group than the mild&moderate OSAS group. Significantly lower FNITBUT, MNITBUT and higher Meiboscore were observed in the severe OSAS subgroup than in the control group, and MNITBUT was higher in severe OSAS objects than in the mild&moderate OSAS objects. LLT and SIT did not exhibit significant differences among control and OSAS subgroups. FNITBUT and MNITBUT showed significantly negative correlations with BMI, while Meiboscore showed a significant positive correlation with AHI.

**Conclusion:**

Patients with OSAS have a tendence of dry eyes, whereas control subjects do not. This indicates us that the OSAS patients should pay more attention to ocular surface care.

## Introduction

Obstructive Sleep Apnea Syndrome (OSAS) is characterized by the frequent collapse and obstruction of the upper airway during sleep, leading to arousals from sleep with or without oxygen desaturation (1, 2). According to a study, OSAS has a prevalence of 30% in white individuals, 32% in black individuals, 38% in Hispanics, and 39% in Chinese individuals (3).

OSAS is a significant risk factor for systemic diseases and multi-organ dysfunction. Recent research has found a correlation between OSAS and eye disorders, such as senile cataract, non-arteritic anterior ischemic optic neuropathy (NAION), normal tension glaucoma (NTG), conjunctival hyperemia, retinal ischemia, and dry eye (4, 5).

Dry eye is a multifactorial disease affecting the ocular surface, marked by ocular symptoms such as tear film instability, hyperosmolarity, ocular surface inflammation, and neurosensory abnormalities (6). Predisposing risk factors for dry eyes and abnormal ocular surface parameters include: Certain medications, Contact lens wear, Systemic diseases, Hormonal changes, Environmental factors, Certain eye surgeries and so on. Some theories argue that OSA-induced oxidative stress can upregulate inflammatory factors, cytokines, and adhesion molecules, leading to local inflammation. This may result in dysfunction of the lacrimal and meibomian glands, causing aqueous-deficient and evaporative dry eye syndrome (DES), respectively (7, 8). Patients with high scores in the Apnea-Hypopnea Index (AHI) are often prone to eye surface inflammation and increased mechanical stress on eye tissues due to the use of continuous positive airway pressure (CPAP) or nasal mask therapy (NMT) devices, leading to a vicious cycle of eye damage (9). Recent studies have reported that OSAS is linked to decreased Schirmer and tear film break-up time (t-BUT) values, indicating a potential tendency toward DES in OSAS patients (10–12). However, ocular surface parameters have not been comprehensively and systematically evaluated, and important clinical indices such as partial blink rate (PBR) have not been investigated in previous studies. To our knowledge, our study is the first comprehensive investigation into OSAS patients’ ocular surface parameters, including PBR. Our research aimed to determine whether there were changes in ocular surface parameters, including PBR, in OSAS patients, and to explore whether these changes were correlated with OSAS severity.

## Materials and methods

### Participants

We recruited all OSAS patients from the otolaryngology department at our hospital. Control subjects were healthy volunteers at the hospital who were confirmed as free of OSAS by an otolaryngologist. The study was approved by the Institutional Review Board of the Second Hospital of Shandong University (China approval number: KYLL-2022-461) and all ethical guidelines of the Declaration of Helsinki were followed. Written informed consent was obtained from all participants prior to the study.

All participants underwent the following examinations in the specified order: standard overnight polysomnography (PSG) and ocular surface assessments. Participants with any of the following conditions were excluded: (1) age under 18 years; (2) use of contact lenses, presence of eyelid or ocular surface diseases, or prior ocular surgery; (3) presence of ocular surface infection, allergy, or systemic autoimmune disease requiring topical ocular medications; (4) history of intraocular surgery, including laser therapy and refractive surgery; (5) history of diabetes mellitus or neurodegenerative diseases; (6) insufficient cooperation during the examinations. Data from both eyes were collected for each participant.

### Sleep study

All participants, including OSAS patients and control subjects, underwent PSG, which included assessments of electroencephalogram (EEG), electro-oculogram, oxygen saturation, electrocardiogram, nasal airflow, thoracic and abdominal inductive plethysmography, and other parameters and pathological events.

Hypopnea was defined as *a* ≥ 30% reduction in nasal pressure flow associated with *a* ≥ 3% desaturation or an arousal (13). The Apnea-Hypopnea Index (AHI, times/h) was calculated based on the average number of episodes of apnea and hypopnea per hour of sleep. Severity of OSAS in patients was categorized into mild (AHI 5–14), moderate (AHI 15–29), or severe (AHI ≥30) based on the criteria set by the American Academy of Sleep Medicine (14). And the normal controls had an AHI of less than 5.

### Assessments of ocular surface

All participants underwent a series of comprehensive clinical evaluations in the ophthalmology department, which included the measurement of axial length (AL), best-corrected visual acuity (BCVA), intraocular pressure (IOP), and slit-lamp biomicroscopy. Following these, ocular surface evaluations were conducted in a specific order, commencing with the measurement of the height of tear meniscus (TMH), first non-invasive tear break-up time (FNITBUT), the mean non-invasive tear break-up time (MNITBUT), the score of Meibomian gland dropout area (Meiboscore), the Schirmer I tear test undertaken without anesthesia (SIT), corneal fluorescein staining (CFS), partial blink rate (PBR), lipid layer thickness (LLT), and ocular surface disease index (OSDI). All ocular surface measurements were conducted by a single, experienced examiner. In the process of our research, we used the same examiner to measure each inspection item using the same machine in order to minimize errors as much as possible. In addition, our examination room is equipped with a thermometer and hygrometer, ensuring that each examination is conducted under the same conditions in order to minimize the impact of environmental temperature and humidity on the subject’s ocular surface.

### OSDI

Each eye was evaluated using the OSDI questionnaire, which assessed ocular surface symptoms. The questionnaire comprised of 12 questions that addressed vision-related symptoms, ocular symptoms, and environmental triggers. Each symptom was graded from 0 to 4. The OSDI score was determined using the formula: OSDI = [(sum of all answers divided by 100)]/[(total number of answers divided by 4)]. Thereby, the OSDI score was calculated, ranging from 0 to 100.

### LLT and PBR

Lipid layer thickness (LLT) and partial blink rate (PBR) were determined in this study using the Lipiview Interferometer (Tear Science Incorporation, Morrisville, NC). Each subject’s tear film interference pattern was documented with a 20 s video, allowing the measurement of interferometric color units for each eye. LLT values up to 100 nm are considered the maximum, and approximately 1 nm of LLT corresponds to one interferometric color unit. PBR measures the ratio of incomplete blinking and total blinking values. These measurements were conducted by the same examiner.

### Tear function

The Keratograph 5 M (Oculus GmbH, Wetzlar, Germany) was used to evaluate tear function, measuring TMH, FNITBUT, MNITBUT, and Meiboscore. Participants were instructed to fixate their gaze on a target, while a Placido disc composed of 22 mire rings was projected onto the corneal surface. A modified tear film scan software was utilized, in conjunction with the Keratograph 5 M, to capture images of the lower tear film meniscus. Three measurements were made, and the mean height was recorded. Infrared photography of the meibomian gland was taken with the same device. The non-invasive break up time analysis was performed with a 5 M keratograph with software for infrared light NIBUT examination. All methods were carried out in accordance with relevant guidelines and regulations. A grid is used to divide the surface of the examined cornea into segments. The first break-up time is the shortest time to the occurrence of the process in the first segment. The mean break-up time is calculated as an arithmetic mean of the break up times in all segments up to a maximum of 24 s of the examination time. The first and mean break up times in all patients were archived in the device’s memory. The first break up time of less than 10 s was considered abnormal. Meiboscores were calculated based on the meibomian gland dropout area, with 0 indicating normal, 1 indicating dropout area <1/3, 2 indicating dropout area <2/3, and 3 indicating dropout area >2/3.

### SIT

The Schirmer I test was undertaken on both eyes simultaneously, without any anesthesia, using Schirmer paper strips (Jingming Limited corporation, Tianjin, China). Participants were asked to keep their eyes closed for 5 min, following which the strips were removed, and the length of the wetting at 5 min was measured.

### CFS

Corneal fluorescein staining is a technique for examining corneal defects and ulcers. Fluorescein sodium was used in combination with a slit-lamp microscope, and the cornea was viewed under blue illumination. The corneal surface was artificially categorized into four parts: the supratemporal, inferotemporal, supernasal, and inferonasal regions. The number of erosions for punctate epithelial was evaluated in each quadrant, and each was scored from 0–3: 0 indicates no epithelial erosion in the cornea, 1 indicates 1–5 dots, 2 indicates 6–30 dots, and 3 indicates >30 dots. The CFS score ranged from 0 to 12 (15).

### Statistical analysis

All statistical analyses were performed using SPSS 21.0 (SPSS, Chicago, IL, United States), and all values were presented as mean ± standard deviation. Generalized estimating equations (GEE) were employed to adjust the correlation between bilateral eyes and compare the clinical basic characteristics and ocular surface parameters among groups. To evaluate the potential for confounding, analyses were also adjusted for age. Only the right eyes of the patients were chosen for Spearman’s correlation coefficient, which was utilized to evaluate the correlations of BMI and AHI with diverse ocular surface parameters. The significance limit was set at 0.05.

Post-hoc power analysis employing G*Power version 3.1.9.7 (Franz Faul, Universitat Kiel, Germany) indicated that a sample size of 142 respondents would provide an 86.4% power with a moderate effect size of 0.28 at a 5% (two-sided) significance level.

## Results

A total of 44 healthy volunteers (88 eyes) and 27 OSAS patients (54 eyes) were ultimately recruited for this study. Of the OSAS patients, 14 had mild or moderate and 13 had severe disease. Table 1 listed the demographic and clinical characteristics of the groups, which revealed no significant difference between OSAS and control subjects concerning age, sex, BCVA, spherical equivalent (SE), or AL. However, the OSAS group had significantly higher AHI and BMI values than the control group.

**Table 1:** Clinical characteristics of the study groups.

	Normal control	OSAS patients		*p*-value^#^
	*n* = 44, 88 eyes	Mild&Moderate	Severe	
		*n* = 14, 28 eyes	*n* = 13, 26 eyes	
Age, years	39.46 ± 21.36	42.43 ± 10.75	37.69 ± 9.14	0.863
Male/female	31/13	9/5	7/6	0.531
BMI	26.60 ± 2.01	29.99 ± 4.38	31.03 ± 5.56	0.006*
AHI	1.77 ± 0.87	16.22 ± 7.85	55.90 ± 17.69	0.000*
BCVA	0.98 ± 0.22	0.86 ± 0.15	0.95 ± 0.11	0.142
IOP, mmHg	13.73 ± 3.21	15.93 ± 3.27	16.17 ± 2.92	0.083
SE, diopters	−0.98 ± 1.06	−1.45 ± 2.19	−1.46 ± 2.30	0.697
AL, mm	23.87 ± 1.15	24.45 ± 0.92	24.05 ± 1.09	0.321

Values are presented as mean ± standard deviation. ^#^Generalized estimating equation among the groups. *Values are statistically significant for *p* < 0.05. BMI, body mass index; AHI, apnea-hypopnea index; ODI, oxygen desaturation index; BCVA, best-corrected visual acuity; IOP, intraocular pressure; SE, spherical equivalent; AL, axial length.

Table 2 displayed ocular surface parameters observed in patients with OSAS and control subjects. FNITBUT, MNITBUT, and TMH were lower (*p* < 0.05 for all) in the OSAS group, while OSDI, CFS, Meiboscore, and PBR were higher than the control group (*p* < 0.05).

**Table 2:** Comparison of ocular surface parameters in OSAS and Control Groups.

	Normal control	OSAS patients	*p*-value^#^
	*n* = 44, 88 eyes	*n* = 27, 54 eyes	
LLT(nm)	81.19 ± 23.21	74.24 ± 27.54	0.109
FNITBUT(s)	8.77 ± 3.99	6.17 ± 4.30	0.000*
MNITBUT(s)	11.83 ± 4.37	10.37 ± 3.76	0.044*
OSDI	9.67 ± 8.03	14.40 ± 3.39	0.005*
SIT	9.50 ± 3.66	9.556 ± 3.312	0.929
CFS	0.18 ± 0.39	0.87 ± 0.87	0.000*
TMH(mm)	0.13 ± 0.02	0.11 ± 0.02	0.000*
Meiboscore	1.20 ± 0.67	1.50 ± 0.88	0.034*
PBR(%)	45.47 ± 40.64	72.67 ± 35.49	0.000*

Values are presented as mean ± standard deviation. ^#^Generalized estimating equation between the normal control and OSAS patients. *Values are statistically significant for *p* < 0.05. LLT, lipid layer thickness; FNITBUT, first non-invasive tear break-up time; MNITBUT, mean non-invasive tear break-up time; OSDI, ocular surface disease index; SIT, the tear test of anesthesia-free Schirmer I; CFS, corneal fluorescein staining; TMH, the height of tear meniscus; PBR(%), partial blinks rate.

**Figure 1:**
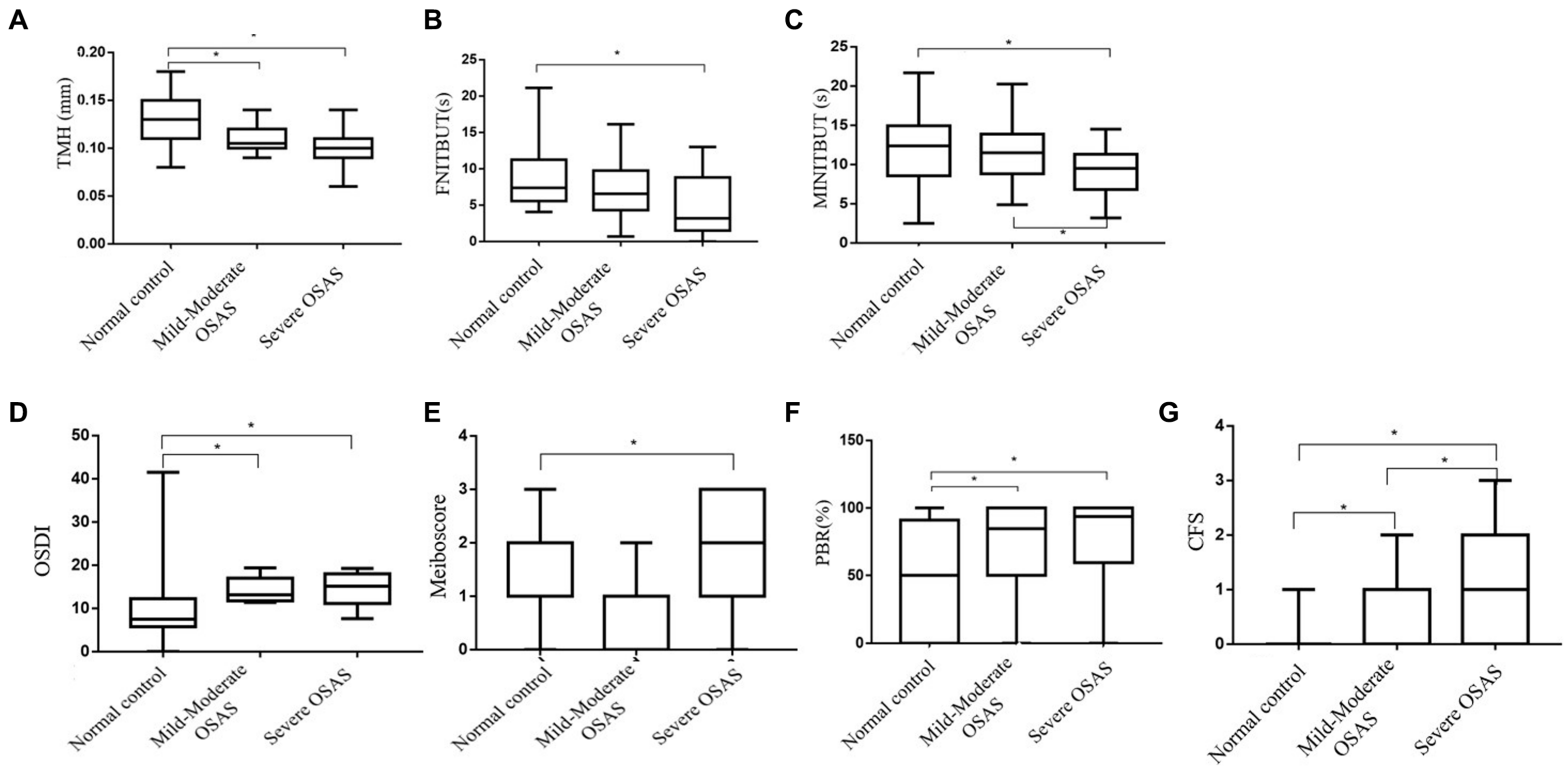
Box-and-whisker plot of the ocular surface parameters in the normal, Mild&Moderate OSAS, and Severe OSAS groups. **(A–G)** Box-and-whisker plot of the TMH, FNITBUT, MINITBUT, OSDI, Meiboscore, PBR, and CFS of each group in the normal, Mild&Moderate OSAS, and Severe OSAS groups. The thick lines represent the means and the error bars represent the standard deviations. The results showed that the Mild&Moderate OSAS group and Severe OSAS group had statistically significant lower TMH than the control group, (*p* < 0.05 or all) and the OSDI, PBR, and CFS in control group were lower than in the Mild&Moderate OSAS group and Severe OSAS group. The CFS was higher in severe OSAS patients than in the mild&moderate OSAS patients (*p* = 0.020). Moreover, the Severe OSAS group had statistically significant higher Meiboscore than the control group (*p* < 0.05). Significantly, lower FNITBUT and MNITBUT were observed in the subjects with severe OSAS than in the control group (*p* < 0.05 for both), and MNITBUT was deeper in severe OSAS patients than in the mild&moderate OSAS patients (*p* = 0.019). TMH, the height of tear meniscus; FNITBUT, first non-invasive tear break-up time; MNITBUT, mean non-invasive tear break-up time; OSDI, ocular surface disease index; PBR(%), partial blinks rate; CFS, corneal fluorescein staining.

Table 3 showed ocular surface parameter measurements of the subgroups. A pairwise comparison between the groups revealed that the mild&moderate and severe OSAS groups had significantly lower TMH and higher OSDI and PBR as compared to the control group (*p* < 0.05 for all), with no significant differences observed between OSAS subgroups. Notably, CFS in the severe OSAS group was higher than that in the mild&moderate OSAS group (*p* < 0.05 for all). Severe OSAS patients had significantly lower FNITBUT, MNITBUT, and higher Meiboscore than the control group (*p* < 0.05 for both), and MNITBUT in severe OSAS patients was lower than that in mild&moderate OSAS patients (*p* = 0.019). No significant differences in LLT and SIT were found between control and OSAS subgroups (*p* > 0.05) (Figure 1).

**Table 3:** Comparison of ocular surface parameters in OSAS subgroups and control groups.

	Normal control	OSAS patients		*p*-value^#^	*p*-value^#^		
	*n* = 44, 88 eyes	Mild&Moderate *n* = 14, 28 eyes	Severe *n* = 13, 26 eyes		1–2	1–3	2–3
	(1)	(2)	(3)				
LLT (nm)	81.19 ± 23.21	74.25 ± 26.01	74.23 ± 29.614	0.278	0.183	0.211	0.998
FNITBUT(s)	8.77 ± 3.99	7.24 ± 4.14	5.02 ± 4.24	0.000*	0.084	0.000*	0.057
MNITBUT(s)	11.83 ± 4.37	11.51 ± 3.89	9.14 ± 3.25	0.014*	0.733	0.004*	0.019*
OSDI	9.67 ± 8.03	14.24 ± 3.03	14.56 ± 3.86	0.020*	0.043*	0.039*	0.808
SIT	9.50 ± 3.66	8.96 ± 3.14	10.192 ± 3.43	0.439	0.494	0.400	0.176
CFS	0.18 ± 0.39	0.61 ± 0.63	1.15 ± 1.01	0.000*	0.000*	0.000*	0.020*
TMH (mm)	0.13 ± 0.02	0.11 ± 0.01	0.10 ± 0.02	0.000*	0.000*	0.000*	0.154
Meiboscore	1.20 ± 0.67	1.36 ± 0.68	1.65 ± 1.06	0.034*	0.300	0.014*	0.222
PBR(%)	45.47 ± 40.64	72.09 ± 33.79	73.03 ± 37.89	0.000*	0.002*	0.002*	0.901

Values are presented as mean ± standard deviation. ^#^Generalized estimating equation among the groups. *Values are statistically significant for *p* < 0.05. LLT, lipid layer thickness; FNITBUT, first non-invasive tear break-up time; MNITBUT, mean non-invasive tear break-up time; OSDI, ocular surface disease index; SIT, the tear test of anesthesia-free Schirmer I; CFS, corneal fluorescein staining; TMH, the height of tear meniscus; PBR(%), partial blinks rate.

**Figure 2:**
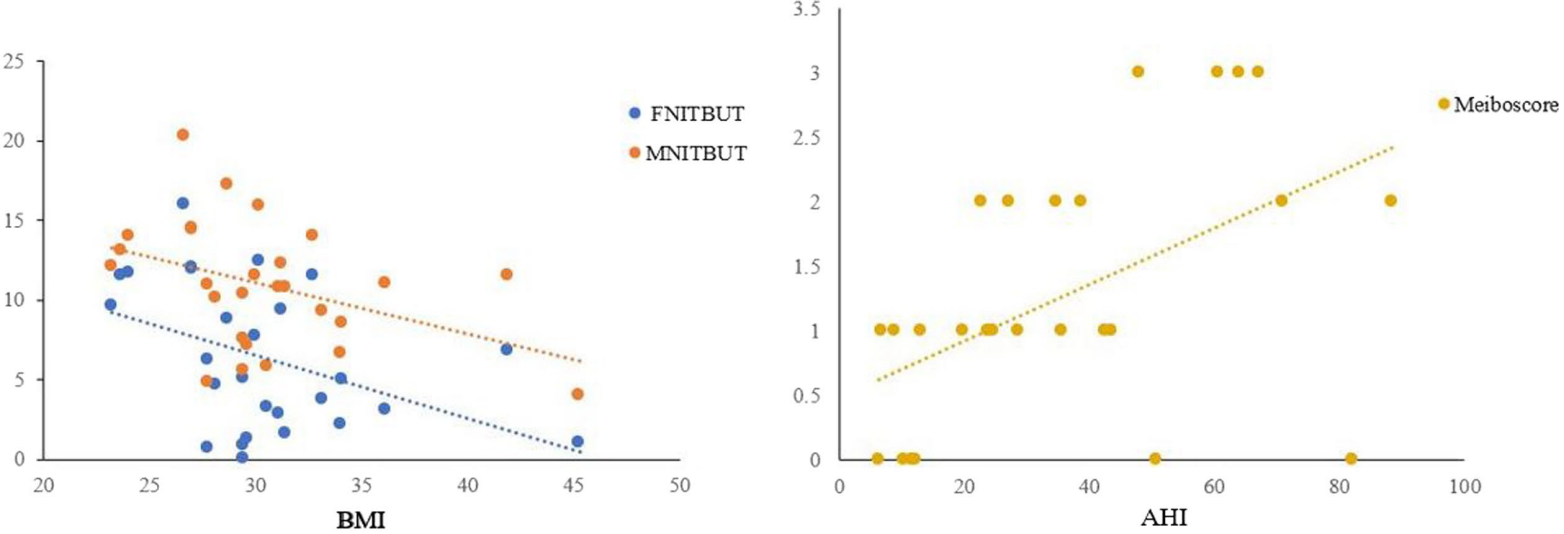
Correlations between FNITBUT, MNITBUT, Meiboscore and BMI and AHI in OSAS eyes. The long thin line represents equivalence. The results showed that significant negative correlations emerged between FNITBUT, MNITBUT, and BMI. But the Meiboscore had a significant positive correlation with AHI (*p* < 0.05). FNITBUT, first non-invasive tear break-up time; MNITBUT, mean non-invasive tear break-up time; BMI, body mass index; AHI, apnea-hypopnea index.

The results of Spearman’s correlation analysis indicated a significant negative correlation between FNITBUT, MNITBUT and BMI (*p* < 0.05 for both), while Meiboscore showed a significant positive correlation with AHI (*p* < 0.05) (Table 4 and Figure 2).

**Table 4:** Correlations between clinical parameters and ocular surface parameters in OSAS.

	BMI		AHI	
	*r*	*p*	*r*	*p*
LLT (nm)	−0.212	0.288	0.110	0.586
FNITBUT(s)	−0.433	0.024*	−0.263	0.185
MNITBUT(s)	−0.399	0.039*	−0.303	0.125
OSDI	0.129	0.523	0.013	0.949
SIT	0.216	0.279	−0.077	0.703
CFS	0.135	0.503	0.282	0.154
TMH (mm)	−0.118	0.557	−0.369	0.058
Meiboscore	0.194	0.331	0.542	0.003*
PBR	0.009	0.966	−0.071	0.726

*r*, Spearman’s correlation coefficient. *Values are statistically significant for *p* < 0.05. LLT, lipid layer thickness; FNITBUT, first non-invasive tear break-up time; MNITBUT, mean non-invasive tear break-up time; OSDI, ocular surface disease index; SIT, the tear test of anesthesia-free Schirmer I; CFS, corneal fluorescein staining; TMH, the height of tear meniscus; PBR(%), partial blinks rate.

## Discussion

Obstructive sleep apnea (OSA) is responsible for more than 85% of sleep apnea (SA) cases (16) and has many adverse effects on the body, which includes the development of various eye complications (as supported by several studies) (4, 17). Dry eye is one of adverse effects associated with OSA according to the literature (9, 18). OSA also contributes to the occurrence of dry eye, which is characterized by corneal and conjunctival epithelial disorders, decreased vision, and Meibomian gland dysfunction. DES is a complex and dynamic ocular surface disease with a prevalence ranging from 5 to 50% (19). In OSA patients, the Schirmer value decreases, indicating aqueous-deficient DES (2). However, few studies have investigated dry eye-related indicators in OSAS patients. Hence, our study aimed to comprehensively evaluate ocular surface indicators, including LLT and PBR, in this population, which is rarely seen in previous studies.

The OSDI questionnaire has become a standard tool for diagnosing and assessing symptoms of dry eye syndrome (20). In our study, the OSDI scores in mild & moderate and severe OSAS groups were significantly higher (*p* < 0.05) compared to the control group. Similar findings were reported in previous studies by Jian Sun et al. (21), Qi Pu et al. (22), and Mutlu Acar et al. (9), who found that severe OSAS cases had high scores in OSDI questionnaire. Another study found a positive correlation between high OSDI scores and low TBUT (9). Consistent with earlier studies, we found that OSAS, particularly the severe forms, was associated with significantly reduced FNITBUT and MNITBUT, implying excessive tear evaporation, relative to controls (*p* < 0.005). Other studies found that OSAS, particularly the moderate and severe forms, was associated with low SIT and TBUT values (9, 22–24). Moreover, we also observed that OSAS was associated with declining TMH levels, which indicated a deficiency of aqueous and propensity towards DES.

Moreover, patients with OSAS tend to have an increased Meiboscore, indicating that morphological changes such as ductal thinning, distortion, and dilatation of Meibomian glands are more common. Previous case-control studies showed that OSAS patients had a greater loss of Meibomian glands in both upper and lower eyelids than controls, and such loss significantly correlates with the severity of OSAS (10, 22, 24). In this population, meibomian gland loss leads to a greater risk of lipid-deficient dry eye. Our study further revealed that patients with OSAS had higher PBR values, a first-in-kind investigation into the relationship between OSAS and eyelid blinking patterns. Numerous factors, including ocular surface disorders, psychological states, and systemic illness, may impact the blink rate (25, 26). Accurate blinking plays a vital role in preserving ocular surface moisture, maintaining the lipid layer’s unity, and extending the lipids in the tear film (27). Inadequate lipid distribution may occur with a rise in partial blinking, leading to increased evaporation (28). Kim (29) also demonstrated that improving blinking patterns can lead to relief from dry eye symptoms and bring about some changes in tear film quality indicators. Therefore, partial blinking might be linked to more severe damage to the ocular (eye) surface and reduced sensitivity in the cornea. Although we observed that LLT in OSAS groups was thinner than in normal controls, this failed to reached statistical significance, likely due to the limited sample size of our study. Appropriate blinking and sustaining tear film stability are essential for preserving ocular health (28). It is widely recognized that increased evaporation due to a deteriorated lipid layer is among the most common causes of hyperosmolarity of the tear film.

Our study also reported significant differences in CFS between the control and OSAS groups, and between mild–moderate and severe OSAS patients. These findings suggested that the corneal epithelium of OSAS patients was inferior to that of normal individuals, which was corroborated by several studies (22, 30, 31). In a previous investigation of the sleep status and dry eye-related risks of OSAS patients, the oxford corneal staining score was higher in OSAS patients with poor sleep quality than those with good sleep quality, with significant staining differences observable between the two groups. Though not the same as our observation index, i.e., corneal fluorescence staining, both measures indicate the ocular surface health of patients (22). Wu et al. (30) also reported significant CFS differences between dry eye patients and controls, with higher scores of dry eye patients as compared to the control group, which supports the findings of our study.

Additionally, there were notable adverse associations established between BMI and TBUT parameters, whereby increasing BMI had a significant impact on decreasing FNITBUT and MNITBUT. Karaca et al. (32) also discovered similar negative correlations between TBUT and both BMI and AHI. However, it should be noted that CFS displayed an opposing negative correlation with BMI. Furthermore, Ran Hao (33) revealed that leptin concentration in tears was negatively interrelated with BMI and as a pro-inflammatory factor, leptin accumulates on the ocular surface, further hastening the process of corneal epithelial cells regeneration (34, 35), promoting ocular fibroblast proliferation and aiding in collagen synthesis (35). These processes assist in repairing ocular damage, thereby mitigating dry eye symptoms and indications (33). OSAS patients with a high BMI may possess a lower concentration of leptin in tears and hence, lesser protection for their ocular surface. In the future, the specific mechanism about leptin concentration in tears plays in OSAS patients is still explored. Nonetheless, Meiboscore showed a significant positive correlation with AHI no significant correlations between ocular surface parameters and AHI were discovered. The contradictions between the results of our study and previous research may arise from various reasons: firstly, dissimilarities in sample characteristics, including ethnicity and age (31). Secondly, the sample size was limited, as the study was conducted solely at a tertiary medical center, where a larger proportion of patients with severe OSAS were included in comparison to other severities. Thus, further investigations concerning subgroups matched for age and sex, with distinct severities of OSAS, are required.

There were several limitations in our study. Firstly, the cross-sectional design of our study did not allow us to establish causality between OSAS and ocular surface parameters. Secondly, the sample was dominated by males, which is reflective of the higher incidence of OSAS in males as compared to females. Thirdly, the statistical analysis of two eyes from the same patient could be seen as a methodological limitation. Additionally, the small sample size is also a limiting factor in our study. Therefore, future research should focus on expanding the sample size to achieve more robust conclusions. Nevertheless, we believe that the findings of our study have significant implications for the treatment of OSAS patients by ophthalmologists. Greater investigations involving larger sample sizes, prospective multicenter studies, and long-term follow-ups should be conducted to confirm our findings.

## Conclusion

There is a propensity for patients with OSAS to develop dry eyes, and clinicians should be cognizant of this possibility.

## Data availability statement

The raw data supporting the conclusions of this article will be made available by the authors, without undue reservation.

## Ethics statement

The studies involving humans were approved by Institutional Review Board of the Second Hospital of Shandong University. The studies were conducted in accordance with the local legislation and institutional requirements. The participants provided their written informed consent to participate in this study. Written informed consent was obtained from the individual(s) for the publication of any potentially identifiable images or data included in this article.

## Author contributions

LH: funding acquisition, methodology, writing – original draft, writing – review & editing, formal analysis, and resources. QT: writing – original draft, writing – review and editing, formal analysis, and investigation. SL and ZX: writing – review and editing. LY: conceptualization, supervision, funding acquisition, writing – original draft, and writing – review and editing. All authors contributed to the article and approved the submitted version.

## Conflict of interest

The authors declare that the research was conducted in the absence of any commercial or financial relationships that could be construed as a potential conflict of interest.

## Publisher’s note

All claims expressed in this article are solely those of the authors and do not necessarily represent those of their affiliated organizations, or those of the publisher, the editors and the reviewers. Any product that may be evaluated in this article, or claim that may be made by its manufacturer, is not guaranteed or endorsed by the publisher.
